# Rapid-Onset Temporal Encephalitis With Negative Cerebrospinal Fluid Polymerase Chain Reaction Testing

**DOI:** 10.7759/cureus.34448

**Published:** 2023-01-31

**Authors:** Davong D Phrathep, Ali El-Husari, Kevin D Healey, Stefan Anthony, Nneoma Onyedimma, Ravish Narvel

**Affiliations:** 1 College of Osteopathic Medicine, Lake Erie College of Osteopathic Medicine, Bradenton, USA; 2 Urology, Lake Erie College of Osteopathic Medicine, Bradenton, USA; 3 Family Medicine, Ascension St. Vincent's Medical Center, Jacksonville, USA; 4 Internal Medicine, Ascension St.Vincent's-Riverside, Jacksonville, USA

**Keywords:** temporal encephalitis, herpes simplex virus infection, new-onset seizure, polymerase chain reactions, herpes virus encephalitis

## Abstract

Herpes simplex encephalitis is a rare disease presentation that is usually characterized by its temporal involvement and positive cerebrospinal fluid (CSF) polymerase chain reaction (PCR) for the herpes simplex virus (HSV). HSV PCR has a sensitivity of 96% and specificity of 99%. Even when the test is negative, if clinical suspicion is high, acyclovir therapy should be continued with a repeated PCR within a week. In this case, we report a 75-year-old female patient who presented with signs of hypertensive emergency with rapid deterioration to seizure-like activity on electroencephalogram (EEG) and signs of temporal encephalitis on magnetic resonance imaging (MRI). The patient did not respond to the initial regimen of antibiotics but did show significant clinical response to acyclovir though she had a negative CSF PCR for HSV ten days after the start of her neurological symptoms. In this case, we argue that alternative methods of diagnosis should be considered in cases of acute encephalitis. Our patient had negative PCR but her computerized tomography (CT), EEG, and MRI results pointed to temporal encephalitis caused by HSV.

## Introduction

Herpes simplex virus (HSV) infection is common with 54% of Americans aged 14 to 49 showing HSV-1 seropositivity and 16% demonstrating HSV-2 seropositivity [1]. HSV-1 is the causative agent in the vast majority of HSV encephalitis cases while HSV-2 is less common and typically seen in immunocompromised patients [1]. HSV-1 is the most common cause of sporadic encephalitis with an incidence estimated at 2-4 cases out of 1,000,000 [2]. Primary HSV infection occurs through mucous membranes or damaged skin contact. Then the virus establishes latent infection within sensory neurons remaining dormant in the dorsal root ganglion. The route by which HSV infection of the central nervous system (CNS) occurs is likely via olfactory or trigeminal nerves or via hematogenous dissemination, however, this specific pathophysiology remains unclear [1]. Furthermore, it remains unclear if the cause of HSV encephalitis is primarily from primary or latent reactivation of the virus [1]. Regardless, HSV encephalitis characteristically involves temporal and hippocampal brain regions [2]. All patients with suspected encephalitis and without absolute contraindications should undergo lumbar puncture, however, this should not delay the administration of empiric antimicrobials. HSV encephalitis cerebrospinal fluid (CSF) analysis typically shows normal or slightly elevated opening pressure, moderate lymphocytic pleocytosis, and elevated erythrocytes and protein with a normal CSF glucose. Despite these characteristics, there is considerable variation such as the erythrocyte count may be normal or minimally elevated, and immunocompromised patients can demonstrate a lack of CSF lymphocytic pleocytosis [1]. HSV polymerase chain reaction (PCR) is the diagnostic test of choice and HSV CSF PCR has a very high sensitivity (96%) and specificity (99%) [3]. False-negative HSV PCR results are not uncommon in early HSV encephalitis, thus when clinical suspicion is high, acyclovir should be continued with repeat CSF HSV PCR within 3-7 days [1]. Here, we present the case of a 75-year-old female with temporal encephalitis with negative PCRs, including HSV, cytomegalovirus (CMV), and West Nile Virus. The novelty of this case stems from the fact that although a negative HSV PCR can occur in less than 3 days or greater than 14 days after treatment, this patient had a negative PCR for HSV on day 10 despite having a significant clinical response to acyclovir therapy.

## Case presentation

A 75-year-old female presented to the emergency department (ED) with complaints of a left-sided body tingling sensation. She reported paresthesia of the left face and left arm numbness for the past 10 days prior to admission. At the time of admission, the patient showed signs of hypertensive emergency with a blood pressure of 263/121. The blood pressure was checked two additional times, reading 259/118 and 262/123. Blood pressures in the right and left arm were similar. The patient was immediately given intravenous hydralazine 10 mg. The patient reported no history of stroke, epilepsy, seizures, incontinence, and oral lacerations. She has had a past medical history of hypertension, coronary artery disease, aortic valve stenosis, diabetes mellitus, peripheral vascular disease, and dyslipidemia. While being assessed by the neurologist in the ED, the patient showed tonic-clonic movement. On physical examination, the patient failed to follow commands and was unoriented to her name, time, and location. At this time, the working diagnosis was possible complex partial seizures secondary to a hypertensive emergency.

**Figure 1:**
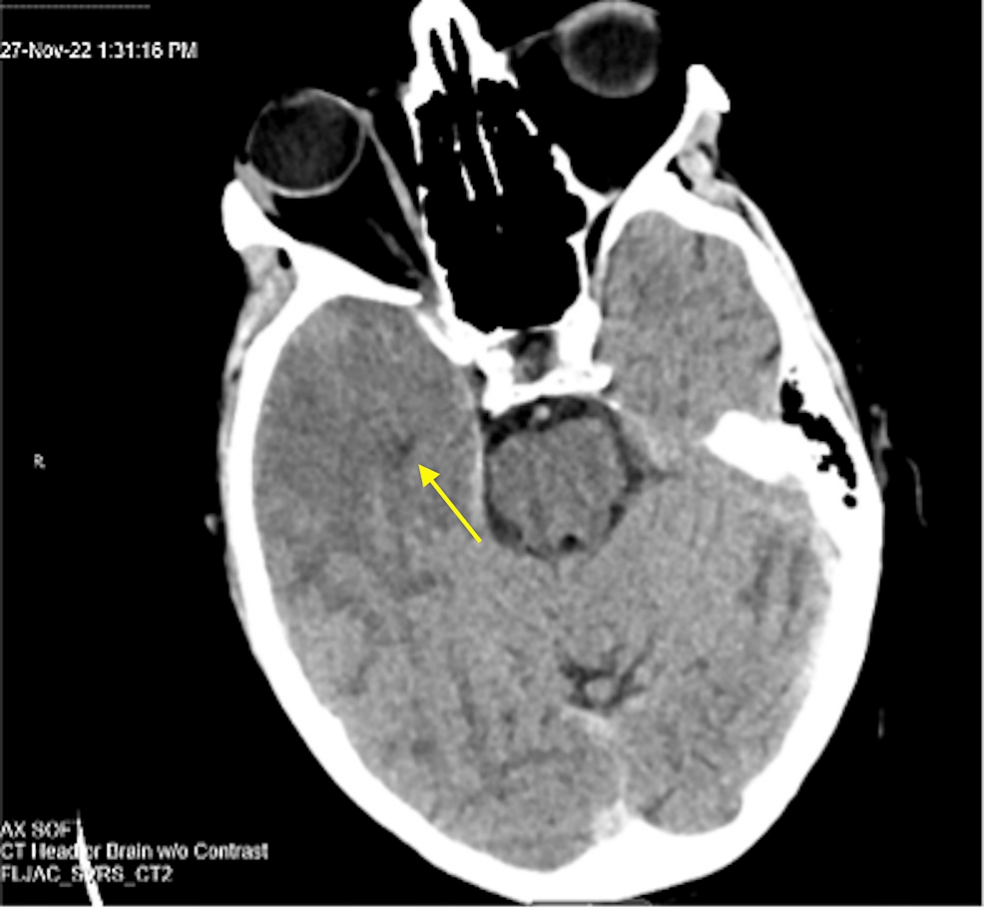
CT head or brain without contrast revealed a zone of hypoattenuation within the region of the right temporal lobe.

The patient was immediately started on intravenous (IV) lorazepam 1 mg IV slow push for every 1 hour as needed and a levetiracetam 1,000 mg IV slow push every 12 hours. Computerized tomography (CT) scan of the head or brain without contrast revealed a zone of hypoattenuation within the region of the right temporal lobe (Figure 1).

**Figure 2:**
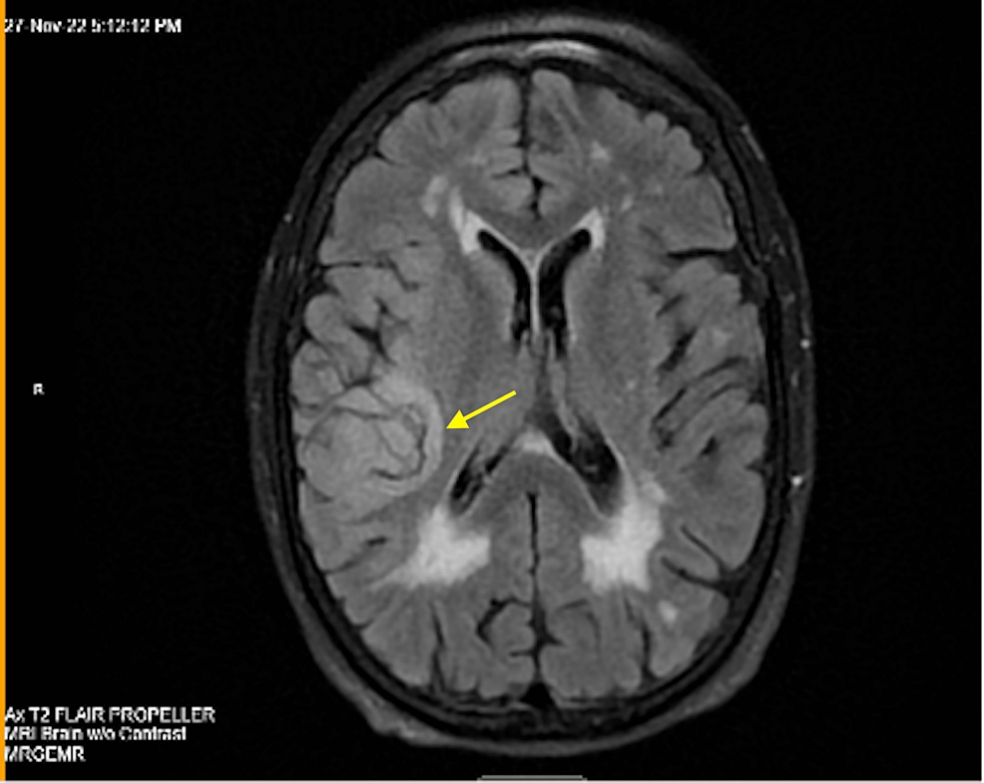
MRI of the brain without contrast revealed cerebral edema, sulcal effacement and cortical diffusion restriction within the right temporal and right posterior parietal regions.

Magnetic resonance imaging (MRI) of the brain without contrast revealed cerebral edema, sulcal effacement, and cortical diffusion restriction within the right temporal and right posterior parietal regions (Figure 2).

The signs were concerning for infarct versus encephalitis. The patient was placed on IV acyclovir 10 mg/kg every 8, IV ceftriaxone 2 g every 12 hours, IV ampicillin 2 g every 6 hours, and IV vancomycin 2 g every 6 hours. Urinalysis and blood cultures were unremarkable. Toxicology screening was unremarkable. As the ED admission progressed, the patient began showing signs of acute hypercapnic respiratory failure, including rapid breathing, confusion, headache, lethargy, and drowsiness. She was transferred to the intensive care unit. While the patient was in the intensive care unit, she began having seizure activity and the patient was immediately intubated for airway protection due to respiratory failure. The patient was sedated with propofol, and her agitation was controlled with IV midazolam 1 mg as needed. Due to the hypertensive emergency, the patient underwent an echocardiogram which revealed a grade 1 diastolic dysfunction with normal left ventricle function, ejection fraction of 55%, and mild aortic stenosis of 1.8 cm^2^. Additionally, the patient was placed on continuous electroencephalogram (EEG) to monitor seizure activity. EEG showed the presence of right frontotemporal and frontocentral focal slowing and occasional sharply contoured waveforms that approximate seizure discharges. At this point, the neurologist believed the condition to be progressive complex partial seizures, status epilepticus related to hypertensive emergency, and right temporal encephalitis. IV sodium valproate 250 mg three times a day was added to improve seizure symptoms.

While the patient was intubated, the patient’s case was discussed with her healthcare surrogate, her son, and he denied the patient’s history of recent travel, insect exposure, and consumption of uncooked meats. The son consented for a lumbar puncture to be done for the patient for the indication of right temporal lobe encephalitis. Fluoroscopic-guided L2-L3 lumbar puncture 1-day post-ED admission which revealed the following CSF analysis (Table 1).

**Table 1:** Cerebrospinal Fluid Analysis

Marker	Patient’s Value	Reference Value
Color - CSF	Colorless	Colorless
Turbidity - CSF	Clear	Clear
Glucose - CSF	102 mg/dL	40-70 mg/dL
Protein - CSF	59 mg/dL	15-45 mg/dL
White Blood Cell Count - CSF	2 cells/mcL	0-5 cells/mm^3^
Red Blood Cell Count - CSF	2 cells/mcL	0-5 cells/mm^3^
Cryptococcal Antigen - CSF	Negative	Negative
Venereal disease research laboratory test - CSF	Non-Reactive	Non-reactive
Herpes Simplex Virus - CSF	Non-Reactive	Non-reactive
Cytomegalovirus - CSF	Non-Reactive	Non-reactive
West Nile Virus - CSF	Non-Reactive	Non-reactive

**Figure 3:**
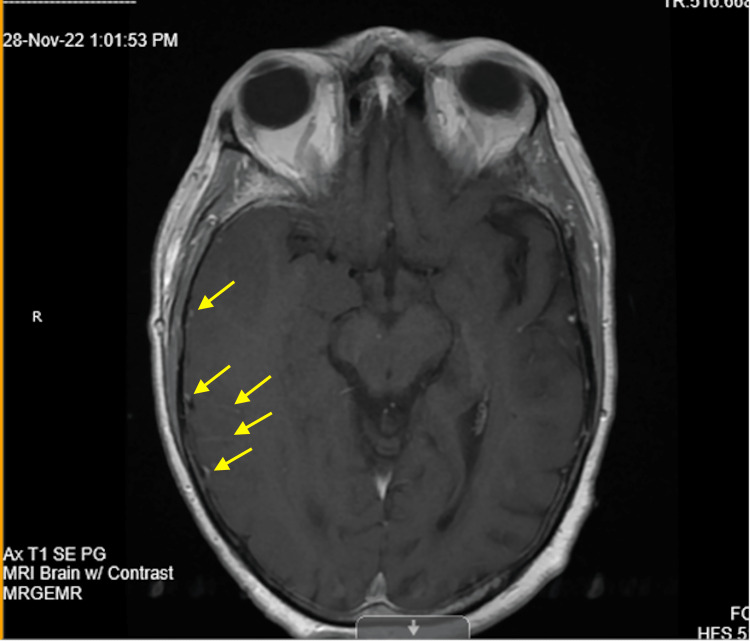
MRI of the brain with contrast revealed increased leptomeningeal and dural enhancement predominantly in the right temporal lobe, which was suspicious for encephalitis.

Paraneoplastic workup, QuantiFERON, and beta-D glucan studies were unremarkable. Despite a negative lumbar puncture for organisms, an MRI of the brain with contrast was ordered. MRI of the brain with contrast revealed increased leptomeningeal and dural enhancement predominantly in the right temporal lobe, which was suspicious for encephalitis (Figure 3).

CT angiography of the head with and without contrast revealed no large branch vessel occlusion, flow-limiting stenosis, or aneurysm in the intracranial arterial system. With high clinical suspicion of herpes simplex encephalitis (HSE), all antibiotics were stopped on day 5 of admission, and acyclovir was advised to be continued. On day 10, the patient began showing signs of improvement due to the continued regimen of IV acyclovir 10 mg/kg every 8 hours. Later that day, she was extubated and was able to follow commands, open her eyes, and speak. She was alert and oriented to name, date of birth, and location but she was unable to recall the details of the event. At this time, it was recommended for the patient to undergo a repeat lumbar puncture to confirm if the cause of her condition was HSV. However, the patient’s son denied repeating lumbar puncture despite medical recommendation. The patient’s son felt that since the patient was getting better, there was no need to put the patient through another procedure to confirm the diagnosis. Despite expert opinion and an attempt at a family conference, the patient’s son was adamant about his decision to not repeat the lumbar puncture. An ethics consultation was not considered due to the patient’s recurrent refusal and the improvement of his mother’s condition with the treatment regimen completed at the hospital. Speech therapy evaluated the patient, and she was placed on a regular diet after passing the modified barium swallow. The patient worked with physical therapy and her condition improved. All IV antibiotics, antivirals, and fluids were discontinued at this time. She was on IV acyclovir 10 mg/kg every 8 hours for 10 days. She was discharged to a short-term rehabilitation facility and the cause of her condition remained unconfirmed but remained highly suspicious for HSV encephalitis.

## Discussion

Encephalitis caused by HSV is a rare event, with an incidence of approximately four cases per million in the United States [4]. With a frequency of one case in 250,000-500,000 people per year, HSE is the most common cause of sporadic viral encephalitis [5]. Without prompt antiviral therapy, approximately 70% of patients with HSE can die [5]. For this reason, rapid and accurate diagnostic procedures are essential for management. The use of PCR techniques to amplify the genome of herpes simplex virus from CSF has become the standard diagnostic procedure of choice [4]. CSF PCR for the herpes virus is highly sensitive and specific for diagnosing herpes encephalitis [6]. The sensitivity and specificity of CSF PCR for HSV exceeds 95%, allowing the exclusion of herpes encephalitis in patients with high pretest probability should the result return negative [7]. Negative CSF HSV PCR tests can occur within the first 72 hours of illness, with subsequent tests becoming positive [4]. Similarly, about 80% of treated patients will have a negative CSF PCR result at greater than 14 days of illness [7]. Thus, CSF HSV PCR was obtained either early before 3 days or late after 14 days since treatment has the potential to result in a negative finding for HSV.

Due to our high suspicion of HSV encephalitis in our patient, we analyze the current literature regarding the potential causes that could have yielded a negative PCR test. Some investigators believe that 1% to 5% of CSF specimens might contain inhibitors that interfere with PCR [8]. For example, a high concentration of porphyrin compounds, which can be derived from the hemolysis of red blood cells, can interfere with PCR reactions [7]. Other explanations for a negative PCR test include the PCR being sent during the first 4 days of neurological symptoms, the viral load being too low for detection, or the CSF sample being too low in volume [9-11]. Low CSF cell counts, location of the infection in the CNS, sample dilution, and variation in primers used during PCR processing have also been suggested as the cause for false-negative results [10,12]. Negative PCR tests tend to also be associated with low CSF protein and leukocyte counts [13]. However, our case demonstrates an atypical presentation with high CSF glucose and protein with normal leukocyte counts. Additionally, our case is unique in the regard that the patient had reportedly been suffering from mild neurologic symptoms for ten days prior to the ED admission. We argue that the admission CSF analysis would’ve yielded a positive result for HSV based on the timeline that the CSF was collected after 72 hours since the onset of neurological symptoms due to infection.

Clinicians should be aware of the drawbacks of CSF PCR testing, specifically false-negative results. Early diagnosis is critical because treatment with acyclovir dramatically decreases morbidity and mortality and can help patients regain independence in activities of daily living [14]. Acyclovir is the only treatment shown to significantly improve prognosis [15]. Although rare, these false negatives can result in premature termination of treatment. The treatment course is weight-based in adults, roughly 10 mg/kg every 8 hours intravenously for 10 to 21 days [6]. Our patient was given acyclovir 750 mg every 12 hours intravenously for 10 days and showed a resolution of her symptoms. It is important to note that patients with HSV encephalitis will typically have a negative CSF HSV PCR after 14 days of acyclovir treatment [6]. A persisting positive PCR should prompt consideration of additional or revised antiviral therapy [4]. Our novel case demonstrates the importance of continuing acyclovir for the improvement of encephalitis despite negative CSF HSV PCR.

With the possibility of false-negative PCR tests, physicians need to use axillary diagnostics like CT, MRI, and EEG to develop a treatment plan immediately due to the poor clinical outcomes of untreated herpes encephalitis. Our case demonstrates how CT, MRI, and EEG can be helpful in diagnosing a patient with a high clinical probability of herpes encephalitis with negative CSF HSV PCR. CT is of limited utility in herpes encephalitis because abnormalities develop late and are nonspecific. Common findings include hypodensity in the temporal and orbital frontal areas [4]. In our case, CT head or brain without contrast revealed a zone of hypoattenuation within the region of the right temporal lobe. Although helpful in supporting the diagnosis of herpes encephalitis, MRI is more sensitive and specific than CT. Because abnormalities appear earlier on MRI, it is the neuroimaging procedure of choice in patients with suspected HSV encephalitis [4]. More than 90% of patients with proven HSV encephalitis will have MRI abnormalities involving the temporal lobes [4]. In our case, the MRI on initial presentation demonstrated increased leptomeningeal and dural enhancement predominantly in the right temporal lobe, which was suggestive for encephalitis. Additionally, MRI demonstrated cerebral edema, sulcal effacement, and cortical diffusion restriction within the right temporal and right posterior parietal regions. To strengthen the diagnosis of herpes encephalitis, EEG may serve as an adjunctive diagnostic and demonstrate periodic lateralized epileptiform discharges as characteristic findings [16]. In our case, EEG showed the presence of right frontotemporal and frontocentral focal slowing and occasional sharply contoured waveforms that approximate seizure discharges. Despite suggestive MRI and EEG and improving clinical symptoms, the patient did not have a repeat PCR due to the son’s refusal against the medical recommendation for repeat lumbar puncture and the final diagnosis of herpes encephalitis could not be confirmed.

Positive EEG, MRI, and CT findings and the improvement of our patient’s condition with IV acyclovir 10 mg/kg every 8 hours for 10 days proved to be the right course of action for our patient. Although the patient was on IV ceftriaxone 2 g every 12 hours, IV ampicillin 2 g every 6 hours, and IV vancomycin 2 g every 6 hours for 5 days, we believed stopping the antibiotic regimen and using acyclovir only was appropriate for the high clinical suspicion for herpes encephalitis and to reconfirm the etiology of the patient’s condition without a repeated lumbar puncture. Because morbidity and mortality increase dramatically as the time to treatment increases [15], we highlight the idea that temporal lobe encephalitis should be treated immediately in the setting of a negative HSV PCR test. The novelty of our case involves the possibility of having negative CSF cultures 72 hours after the onset of symptoms and the importance of interpreting the results in the context of the likelihood of herpes encephalitis. We present an atypical presentation of suspected idiopathic herpes encephalitis with negative CSF PCR tests but supported by positive EEG, MRI, and CT findings, resulting in the resolution of symptoms with empiric acyclovir.

## Conclusions

Even when PCR findings are negative, our case highlights the importance of suspecting herpes encephalitis in all cases of acute encephalitis. In our case, the patient’s PCR was negative after 72 hours whereas suspected HSV encephalitis was expected to yield positive results. Additionally, we were unable to perform a repeat lumbar puncture due to the patient’s son refusing an expert recommendation. Despite no-repeat lumbar puncture and confirmatory repeat HSV PCR, we relied on the positive CT, MRI, and EEG findings to develop a treatment plan for our patient. Here we demonstrated a successful case of improved temporal lobe encephalitis due to empiric acyclovir treatment without confirmed HSV PCR serology in a 75-year-old female.
